# Constructing the prediction model based on DXA between sarcopenia and BMD in middle-aged and elderly men with T2DM

**DOI:** 10.3389/fmed.2025.1655263

**Published:** 2025-08-12

**Authors:** Guoyang Zhang, Lidan Huang, Liangzhong Liao

**Affiliations:** Department of Radiology, Xiamen Hospital of Traditional Chinese Medicine, Xiamen, China

**Keywords:** type 2 diabetes mellitus, sarcopenia, bone mineral density, dual-energy X-ray absorptiometry, prediction model

## Abstract

**Objective:**

To explore the relationship between sarcopenia and bone mineral density (BMD) in middle-aged and elderly male patients with type 2 diabetes mellitus (T2DM), construct a prediction model for sarcopenia based on dual-energy X-ray absorptiometry (DXA), and evaluate its clinical value.

**Methods:**

A total of 523 middle-aged and elderly male patients with T2DM in the hospital from January 2021 to December 2024 were selected and divided into the training set (366 cases) and the validation set (157 cases) at a ratio of 7:3. The BMD *T*-value was measured by DXA, and clinical data were collected. A prediction model was constructed using multivariate logistic regression in the training set, and the model efficacy was evaluated by receiver operating characteristic (ROC) curve, calibration curve, and decision curve analysis (DCA).

**Results:**

The incidence of sarcopenia was 27.05% (99/366) in the training set and 28.02% (44/157) in the validation set. Multivariate analysis showed that age, HbA1c, and HOMA-IR were independent risk factors for sarcopenia, while the lumbar L1–L4 *T*-value, and femoral neck *T*-value were independent protective factors for sarcopenia (*p* < 0.05). The C-index of the nomogram model were 0.773 (in the training set) and 0.750 (in the validation set) respectively. The calibration curve showed good agreement between predicted and actual values, and the Hosmer–Lemeshow test were significant (all *p* > 0.05). The ROC curve showed the area under the curve (AUC) of the nomogram model for predicting the risk of sarcopenia was 0.773 (95% CI: 0.652–0.895) and 0.750 (95% CI, 0.686–0.814) in the training set and the validation set, respectively. The sensitivity and specificity were 0.714, 0.887 and 0.688, 0.796, respectively.

**Conclusion:**

The prediction model constructed based on DXA can effectively predict the risk of sarcopenia in middle-aged and elderly male patients with T2DM, providing a basis for clinical early screening and intervention.

## Introduction

With the intensification of population aging, type 2 diabetes mellitus (T2DM) has become a significant global public health issue (1). Epidemiological studies have shown that the prevalence of T2DM in the middle-aged and elderly population reaches 15–20%. It is often accompanied by sarcopenia and a decrease in bone mineral density (BMD), which seriously affects the quality of life (2). Sarcopenia is characterized by a reduction in muscle mass, a decline in strength, and a decrease in function, while a decrease in BMD is the core marker of osteoporosis. These two conditions often co-exist in middle-aged and elderly T2DM patients, resulting in an “imbalance in muscle-bone interaction” (3). Dual-energy X-ray absorptiometry (DXA), as the gold standard for evaluating muscle mass and BMD, can accurately measure the appendicular skeletal muscle mass (ASM) and the BMD of the lumbar spine, femoral neck, and other parts.

Studies have indicated that sarcopenia and a decrease in BMD in T2DM patients may share pathological mechanisms such as insulin resistance, chronic inflammation, and oxidative stress: insulin resistance inhibits muscle protein synthesis and interferes with the function of osteoblasts; hyperglycemia induces the accumulation of advanced glycation end products (AGEs), which damages the microstructures of muscle and bone; persistent low-grade inflammation [such as an increase in interleukin-6 (IL-6) and tumor necrosis factor-α (TNF-α)] accelerates muscle breakdown and bone resorption (4, 5). However, current studies on middle-aged and elderly male T2DM patients have limitations: the quantitative association between sarcopenia and BMD lacks large-sample support, and a prediction model for sarcopenia based on DXA indicators has not been established (6). As a clinical decision-making tool, a prediction model can integrate multi-dimensional indicators to achieve early quantitative assessment of the risk of sarcopenia. However, the application of previous prediction models that combine muscle mass, BMD, and metabolic indicators in T2DM patients is still in the exploratory stage.

In this study, DXA was used to measure the musculoskeletal indicators of middle-aged and elderly male T2DM patients. The correlation between them was analyzed, and a nomogram prediction model was constructed to provide a basis for early clinical screening and personalized intervention.

## Methods

### Study subjects

A total of 523 middle-aged and elderly male patients with T2DM in Xiamen Hospital of Traditional Chinese Medicine from January 2021 to December 2024 were selected as the research subjects. Patients were randomly allocated to the training set (70%, *n* = 366) and validation set (30%, *n* = 157) using computer-generated random numbers (SPSS 26.0, IBM Corporation, United States). The randomization ensured comparable baseline characteristics between the two sets. This study was approved by the Ethics Committee of Xiamen Hospital of Traditional Chinese Medicine (No. TCM10-1053). This study adhered to the tenets of the Declaration of Helsinki. Written informed consent was obtained from all participants.

### Inclusion and exclusion criteria

Inclusion criteria: (1) meeting the diagnostic criteria for type 2 diabetes (7), (2) age ≥45 years, (3) both the patients and their families signed the informed consent forms.

Exclusion criteria: (1) complicated with other endocrine diseases, (2) having severe heart, lung, liver, or kidney function disorders, (3) recently using drugs that affect bone metabolism or muscle mass, (4) having a history of malignant tumors.

### Collection of baseline data

The patients’ basic information was recorded, including age, height, and weight, and the body mass index (BMI) was calculated. Smoking history and drinking history were also recorded. The detection of diabetes-related indicators included the duration of diabetes, fasting plasma glucose (FPG), 2-h postprandial glucose (2hPG), hemoglobin A1c (HbA1c), and the homeostatic model assessment of insulin resistance (HOMA-IR). For the diagnosis of sarcopenia, the appendicular skeletal muscle (ASM) was measured using a dual-energy X-ray absorptiometer, and the appendicular skeletal muscle mass index (ASMI) was calculated. Grip strength was measured using a dynamometer, and gait speed was evaluated through a 4-meter walking test. The diagnosis of sarcopenia was based on the 2019 criteria of the Asian Working Group for Sarcopenia (AWGS) (8). BMD *T*-values of the lumbar spine (L1–L4) and the femoral neck were measured by DXA. In addition, total cholesterol (TC), triglycerides (TG), high-density lipoprotein cholesterol (HDL-C), low-density lipoprotein cholesterol (LDL-C), and serum creatinine (SCr) were detected, and the estimated glomerular filtration rate (eGFR) was estimated.

### Grouping method

Patients’ BMD and skeletal muscle mass (SMM) were measured using dual-energy DXA. BMD was expressed in g/cm^2^, and SMM was expressed in kg. Sarcopenia diagnosis strictly followed AWGS 2019 criteria requiring: (1) Low muscle mass (ASMI <7.0 kg/m^2^ by DXA) AND (2) Either low muscle strength (grip strength <28 kg) OR low physical performance (gait speed <1.0 m/s) (8). All measurements were performed by trained staff using standardized protocols with daily quality control checks.

### Statistical analysis

Data were analyzed using SPSS 26.0 (IBM Corporation, United States) and R4.5.1 (R Foundation for Statistical Computing, Austria). Continuous variables conforming to the normal distribution were expressed as x̄ ± s; otherwise, they were expressed as M (Q₁, Q₃). Categorical variables were expressed as *n* (%). Patients were divided into the training set and the validation set at a ratio of 7:3. Univariate analysis was performed using the *t*-test, Mann–Whitney *U* test, or *χ*^2^ test, and indicators with *p* < 0.05 were selected and included in the multivariate logistic regression. Multivariate analysis excluded diagnostic components of sarcopenia (ASM/ASMMI), grip strength and gait speed to avoid tautological prediction and collinearity. The nomogram model was constructed using the “rms” package in R language. The model efficacy was evaluated through the receiver operating characteristic (ROC) curve (area under the curve, AUC), calibration curve, C-index, and decision curve analysis (DCA). *p* < 0.05 was considered statistically significant.

## Results

### Comparison of baseline data between the training set and the validation set

The incidence of sarcopenia in the training set was 27.05% (99/366), and that in the validation set was 28.02% (44/157). There were no statistically significant differences in baseline data such as age, gender, smoking history, and BMI between the training set and the validation set (*p* > 0.05), indicating that the two groups had good balance and could be used for subsequent analysis (Table 1).

**Table 1:** Comparison of baseline data between the training set and the validation set.

Indicators	Training set (*n* = 366)	Validation set (*n* = 157)	*t*/*χ*^2^	*p*
Age (years)	66.25 ± 7.31	67.10 ± 7.12	1.228	0.219
Height (m)	1.68 ± 0.07	1.67 ± 0.06	1.560	0.119
Weight (kg)	68.52 ± 8.74	67.94 ± 8.61	0.698	0.485
BMI (kg/m^2^)	22.63 ± 2.52	23.02 ± 2.33	1.658	0.097
Smoking history (yes/no)	223/143	95/62	0.008	0.928
Drinking history (yes/no)	95/271	39/118	0.071	0.788
Disease duration (years)	8.83 ± 3.52	8.52 ± 3.56	0.920	0.358
FPG (mmol/L)	8.31 ± 1.62	8.16 ± 1.52	0.513	0.607
2hPG (mmol/L)	11.82 ± 2.42	11.74 ± 2.13	0.358	0.719
HbA1c (%)	7.92 ± 1.33	7.78 ± 1.25	1.123	0.261
HOMA-IR	2.91 ± 0.81	2.84 ± 0.90	0.875	0.381
ASM (kg)	22.51 ± 3.21	22.13 ± 3.02	1.262	0.207
ASMMI (kg/m^2^)	7.91 ± 0.80	7.81 ± 0.77	1.324	0.185
Grip strength (kg)	29.52 ± 4.22	29.18 ± 4.03	0.855	0.392
Gait speed (m/s)	0.93 ± 0.16	0.91 ± 0.15	1.334	0.182
Lumbar spine L1–L4 *T*-value	−0.82 ± 0.91	−0.91 ± 0.81	1.070	0.284
Femoral neck *T*-value	−0.71 ± 0.82	−0.83 ± 0.72	1.589	0.112
TC (mmol/L)	4.92 ± 0.82	4.83 ± 0.92	1.108	0.286
TG (mmol/L)	1.77 ± 0.62	1.82 ± 0.73	0.800	0.423
HDL-C (mmol/L)	1.22 ± 0.31	1.18 ± 0.31	1.352	0.176
LDL-C (mmol/L)	2.91 ± 0.73	2.86 ± 0.63	0.747	0.455
SCr (μmol/L)	79.23 ± 11.52	78.63 ± 12.32	0.534	0.593
eGFR (mL/min/1.73m^2^)	87.52 ± 9.82	88.13 ± 10.13	0.645	0.519

### Univariate analysis of influencing factors for sarcopenia

Univariate analysis revealed that among the patients in the training set, there were statistically significant differences (*p* < 0.05) in age, HbA1c, HOMA-IR, lumbar L1–L4 *T*-value, ASM, ASMI, and disease duration between the sarcopenia group and the non-sarcopenia group (Table 2).

**Table 2:** Univariate analysis of influencing factors for sarcopenia.

Indicators	Sarcopenia group (*n* = 99)	Non-sarcopenia group (*n* = 267)	*t*/*χ*^2^	*p*
Age (years)	68.52 ± 6.81	66.35 ± 7.21	2.595	0.009
Height (m)	1.67 ± 0.06	1.68 ± 0.07	1.259	0.208
Weight (kg)	65.24 ± 8.12	66.87 ± 8.54	1.643	0.101
BMI (kg/m^2^)	22.32 ± 2.11	22.15 ± 2.34	0.629	0.529
Smoking history (yes/no)	65/34	158/109	1.274	0.259
Drinking history (yes/no)	32/67	63/204	2.862	0.090
Disease duration (years)	10.25 ± 3.87	9.12 ± 3.21	2.824	0.005
FPG (mmol/L)	8.89 ± 1.72	8.52 ± 1.64	1.892	0.059
2hPG (mmol/L)	12.06 ± 2.54	11.52 ± 2.33	1.921	0.055
HbA1c (%)	8.05 ± 1.23	7.70 ± 1.28	2.348	0.019
HOMA-IR	3.15 ± 0.91	2.91 ± 0.79	2.475	0.013
Lumbar spine L1–L4 *T*-value	−1.23 ± 0.84	−0.97 ± 0.89	2.520	0.012
Femoral neck *T*-value	−1.15 ± 0.79	−0.91 ± 0.82	2.511	0.012
TC (mmol/L)	5.03 ± 0.85	4.88 ± 0.80	1.566	0.118
TG (mmol/L)	1.89 ± 0.65	1.78 ± 0.63	1.471	0.142
HDL-C (mmol/L)	1.18 ± 0.32	1.25 ± 0.33	1.817	0.070
LDL-C (mmol/L)	3.02 ± 0.76	2.89 ± 0.72	1.511	0.131
SCr (μmol/L)	80.24 ± 11.86	78.84 ± 11.26	1.041	0.298
eGFR (mL/min/1.73m^2^)	86.21 ± 10.13	88.36 ± 9.54	1.883	0.060

### Multivariate logistic regression analysis of influencing factors for sarcopenia

Sarcopenia was used as the dependent variable, and the factors with statistical significance in the univariate analysis were used as independent variables for multivariate logistic regression analysis (the variable assignments were shown in Table 3). Multivariate analysis excluded diagnostic components of sarcopenia (ASM/ASMMI), grip strength and gait speed to avoid tautological prediction and collinearity. The results showed that age, HbA1c, and HOMA-IR were independent risk factors influencing sarcopenia (*p* < 0.05), while the lumbar spine L1–L4 *T*-value, and femoral neck *T*-value were independent protective factors influencing sarcopenia (*p* < 0.05) (Table 4).

**Table 3:** Variable assignment methods.

Variable	Meaning	Assignment
X1	Age	Continuous variable
X2	Disease duration	Continuous variable
X3	HbA1c	Continuous variable
X4	HOMA-IR	Continuous variable
X5	Lumbar spine L1–L4 *T*-value	Continuous variable
X6	Femoral neck *T*-value	Continuous variable
Y	Sarcopenia	1 = Sarcopenia, 0 = Non-sarcopenia

**Table 4:** Logistic regression analysis of influencing factors for sarcopenia.

Indicators	*β*	SE	Wald	*p*	OR	95% CI
Age	0.039	0.018	4.630	0.031	1.040	1.004–1.07
Disease duration	0.083	0.040	4.343	0.057	1.086	0.955–1.175
HbA1c	0.259	0.108	5.735	0.017	1.296	1.048–1.601
HOMA-IR	0.406	0.158	6.595	0.010	1.501	1.101–2.047
Lumbar spine L1–L4 *T*-value	−2.252	0.436	26.700	0.001	0.105	0.045–0.247
Femoral neck *T*-value	−1.784	0.457	15.231	0.001	0.168	0.069–0.412

### Establishment of a prediction model for sarcopenia in middle-aged and elderly men with type 2 diabetes mellitus

**Figure 1:**
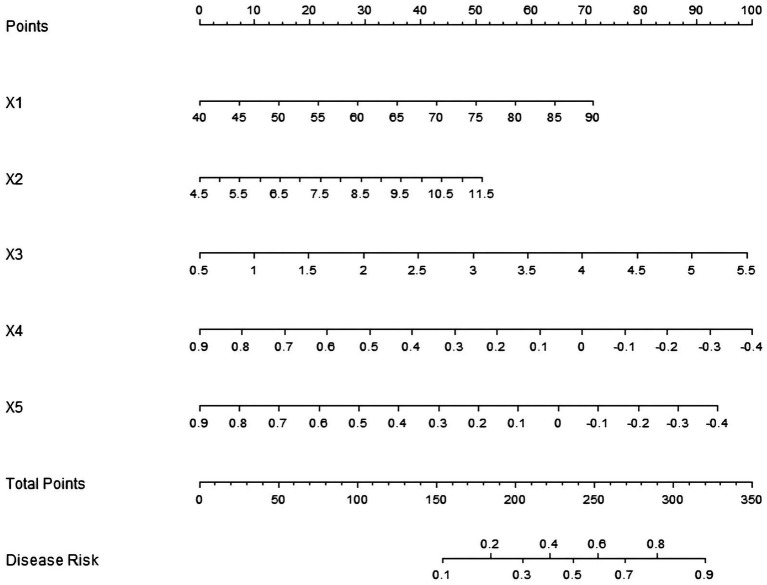
Establishment of a prediction model for sarcopenia in middle-aged and elderly male patients with T2DM. X1: age; X2: HbA1c; X3: HOMA-IR; X4: lumbar spine L1–L4 *T*-value; X5: femoral neck *T*-value.

Based on the influencing factors determined by multivariate logistic regression analysis, a nomogram model for predicting sarcopenia in middle-aged and elderly men with T2DM was constructed. In this model, scores were assigned to each independent influencing factor, and the probability of sarcopenia was predicted by calculating the total score. The higher the total score, the higher the probability of predicting sarcopenia in middle-aged and elderly men with type 2 diabetes mellitus (Figure 1).

### Evaluation and validation of the prediction model for sarcopenia in middle-aged and elderly male patients with T2DM

**Figure 2:**
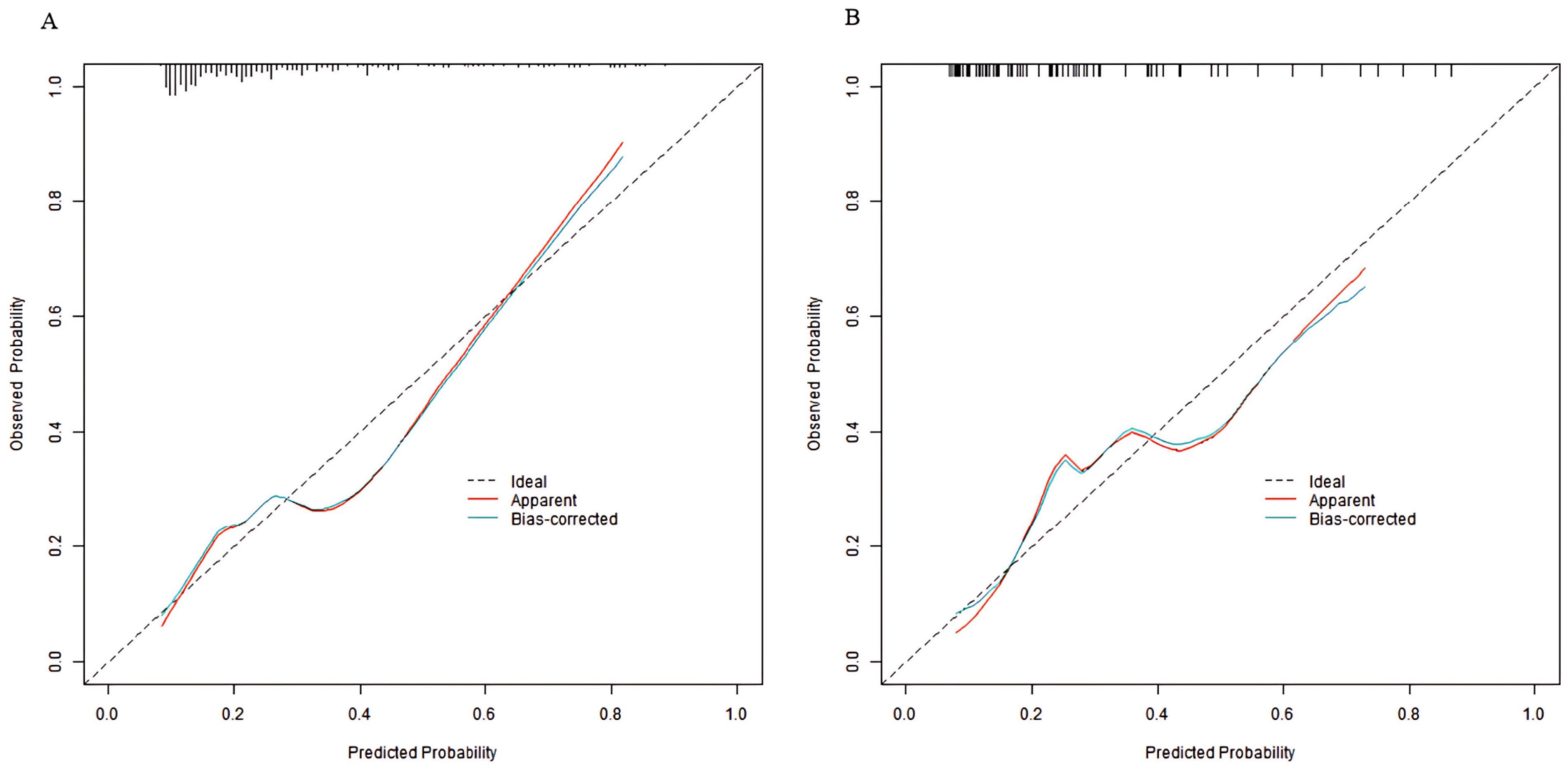
Calibration curves in the training set **(A)** and the validation set **(B)**.

**Figure 3:**
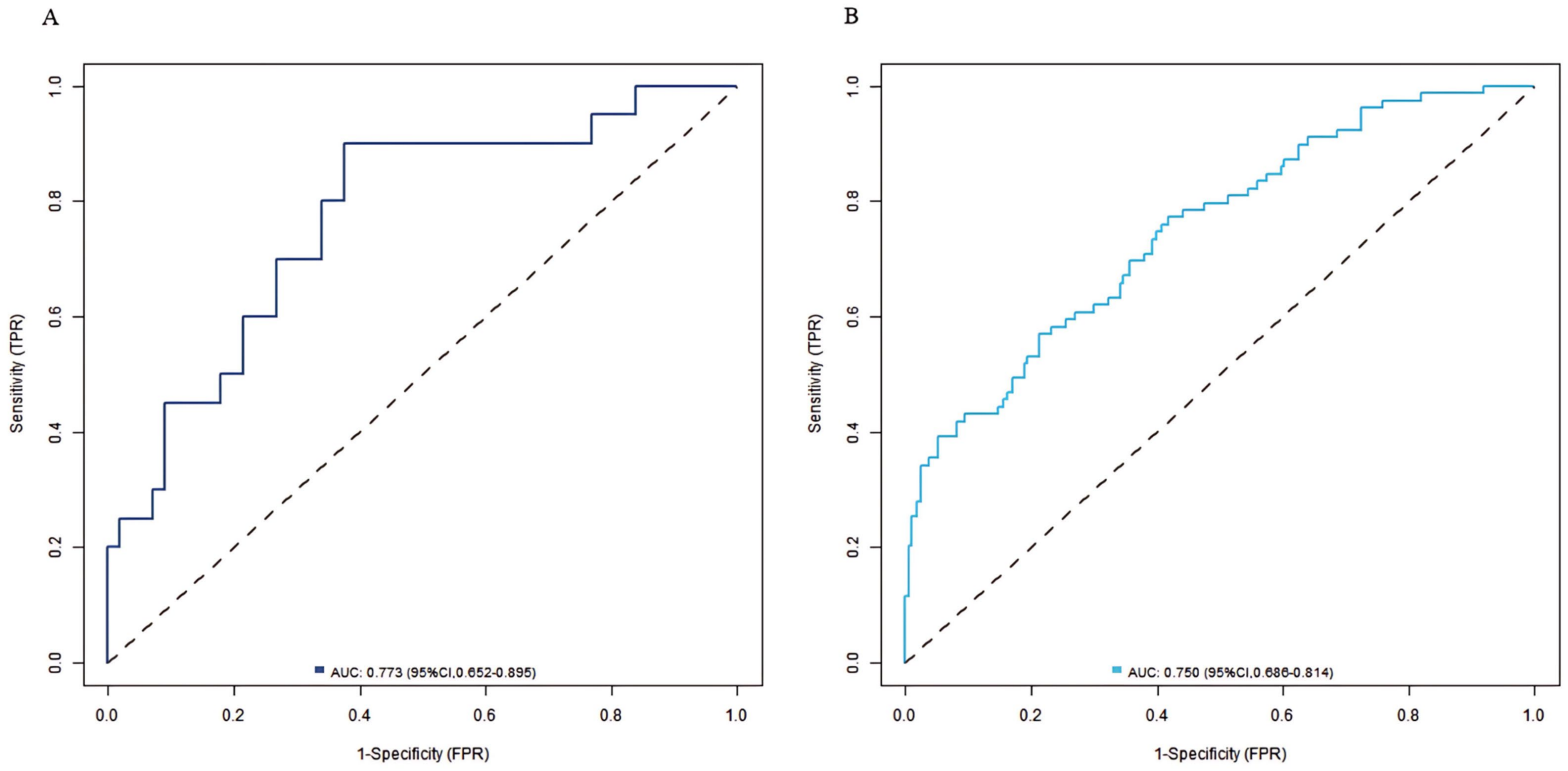
ROC curves in the training set **(A)** and the validation set **(B)**.

In the training set and validation set, the C-index of the nomogram model was 0.773 and 0.750, respectively. The calibration curve showed a good agreement between the predicted values and the actual values. The results of the Hosmer–Lemeshow test were *χ*^2^ = 10.955 (*p* = 0.204) and *χ*^2^ = 8.139 (*p* = 0.420), respectively. The ROC curves showed that in the training set and validation set, the AUCs of the nomogram model were 0.773 (95% CI: 0.652–0.895) and 0.750 (95% CI: 0.686–0.814), respectively. The sensitivity and specificity were 0.714, 0.887 and 0.688, 0.796, respectively. The calibration curves were shown in Figure 2, and the ROC curves were shown in Figure 3.

### Decision curve analysis of the prediction model for sarcopenia in middle-aged and elderly men with type 2 diabetes mellitus

**Figure 4:**
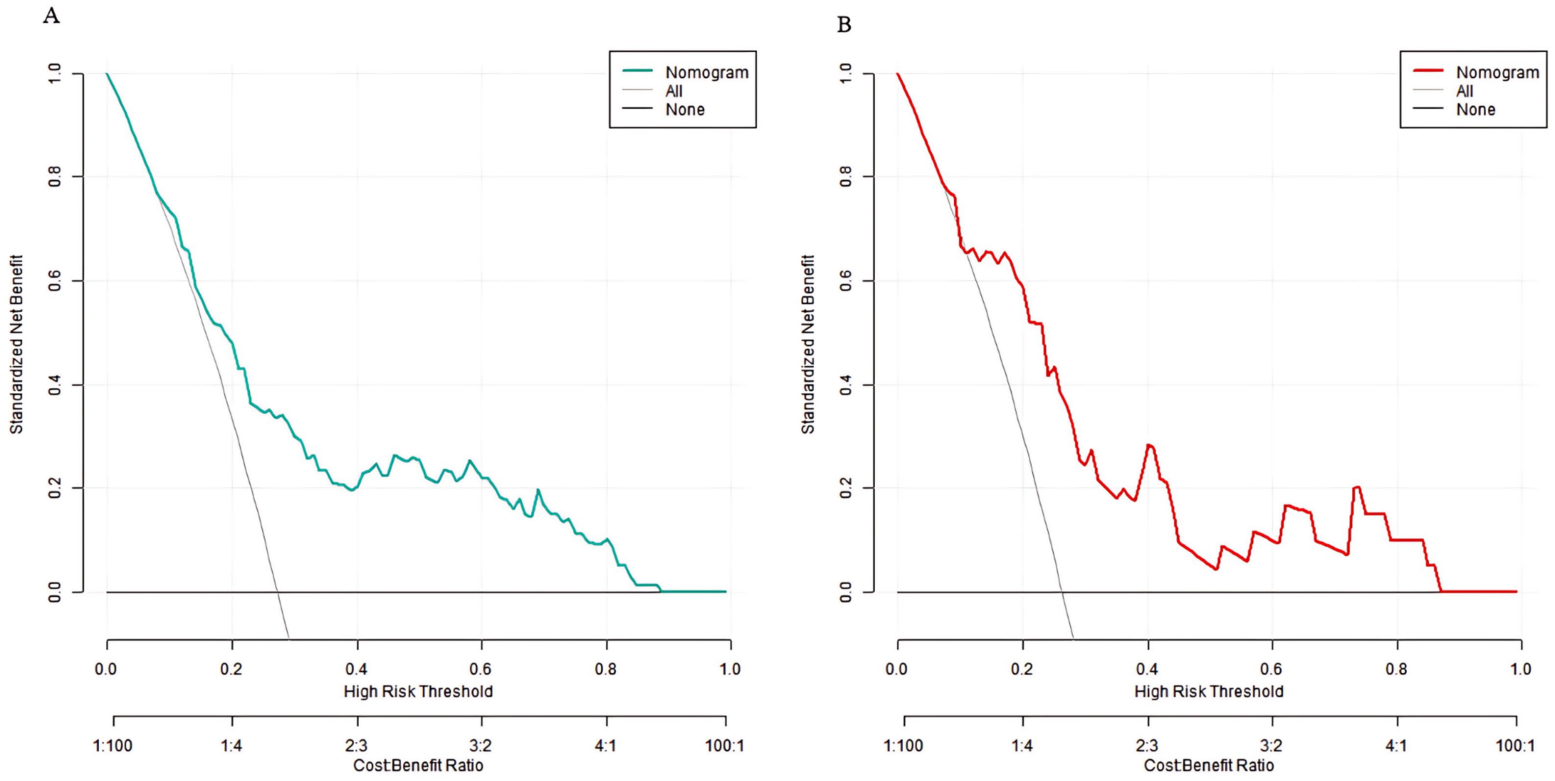
Decision curves in the training set **(A)** and the validation set **(B)**.

Decision curve analysis showed that when the threshold probability was between 0.06 and 0.92, the decision of applying the nomogram model constructed in this study to predict sarcopenia in middle-aged and elderly men with type 2 diabetes mellitus had more clinical benefits compared with the decisions of pre-operatively assuming that all patients had sarcopenia or none of them had sarcopenia (Figure 4).

## Discussion

In this study, dual-energy X-ray absorptiometry (DXA) was employed to analyze the relationship between sarcopenia and BMD in middle-aged and elderly men with type 2 diabetes mellitus (T2DM), and a risk prediction model for sarcopenia based on multivariate logistic regression was constructed. The results indicated that age, HbA1c, and HOMA-IR were independent risk factors for sarcopenia, while the *T*-scores of lumbar vertebrae L1–L4, the *T*-score of the femoral neck, appendicular skeletal muscle mass (ASM), and appendicular skeletal muscle mass index (ASMI) were independent protective factors. The C-indexes of the nomogram model were 0.773 and 0.750, and the AUCs of the ROC curves were 0.773 and 0.750. Decision curve analysis (DCA) showed that it had clinical application value, providing a basis for early clinical screening and intervention.

Multivariate logistic regression analysis revealed that age was an important independent risk factor for sarcopenia. With the increase of age, various physiological functions of the human body gradually decline, and muscle tissue is no exception (9). During the aging process, the balance between muscle protein synthesis and decomposition is disrupted, with a decrease in synthesis and an increase in decomposition, leading to a gradual reduction in muscle mass. This phenomenon may be more obvious in diabetic patients. Diabetes itself accelerates the aging process and affects muscle metabolism through multiple mechanisms such as insulin resistance and oxidative stress, making elderly diabetic patients more prone to sarcopenia (10, 11). In this study, for every 1-year increase in age, the risk of sarcopenia increased by 4.4%, which was consistent with the results of many previous studies, further verifying the important role of age in the occurrence and development of sarcopenia (12).

An elevated HbA1c level was also an independent risk factor for sarcopenia (13). HbA1c reflects the average blood glucose level of patients in the past 2–3 months, and its increase indicates poor blood glucose control (14). Hyperglycemia can damage muscle health through multiple pathways. Persistent hyperglycemia induces the production of advanced glycation end-products (AGEs), which can bind to proteins in muscle tissue, altering their structure and function, leading to muscle fibrosis and functional decline. In this study, for every 1% increase in HbA1c, the risk of sarcopenia increased by 30.1%, suggesting that good blood glucose control is crucial for the prevention of sarcopenia. HOMA-IR, as an important indicator for evaluating insulin resistance, was also closely associated with an increased risk of sarcopenia when elevated (15). In the state of insulin resistance, the effect of insulin in promoting muscle protein synthesis is weakened, and it also interferes with the transport and utilization of amino acids, resulting in a decrease in muscle mass (16). In addition, insulin resistance can further inhibit muscle growth by affecting the function of the growth hormone-insulin-like growth factor-1 (GH-IGF-1) axis (17). The results of this study showed that for every 1-unit increase in HOMA-IR, the risk of sarcopenia increased by 52.1%, highlighting the importance of improving insulin resistance in the prevention of sarcopenia. Although our study did not measure inflammatory biomarkers directly, the observed association between elevated HbA1c/HOMA-IR and sarcopenia aligns with prior evidence linking hyperglycemia and insulin resistance to muscle deterioration (18). Specifically, our multivariate analysis identified HbA1c and HOMA-IR as independent risk factors for sarcopenia (*p* < 0.05), which may indirectly support the involvement of metabolic dysregulation in muscle loss. Future studies incorporating inflammatory markers (e.g., IL-6, CRP) are needed to validate these mechanistic pathways in T2DM patients.

There was a significant negative correlation between the *T*-scores of BMD of lumbar vertebrae L1–L4 and the femoral neck and sarcopenia, indicating that higher BMD was a protective factor for sarcopenia (19). There is a close “muscle-bone interaction” relationship between bone and muscle. The mechanical load generated by muscle contraction is an important factor in maintaining BMD, and bones also provide attachment points and support for muscles. When muscle mass decreases, the mechanical stimulation to bones weakens, which can lead to a decrease in BMD. Conversely, higher BMD may reflect better muscle function and load status of the body (20, 21). In this study, for every 1-unit increase in the *T*-score of lumbar vertebrae L1–L4, the risk of sarcopenia decreased by 88.8%, and for every 1-unit increase in the *T*-score of the femoral neck, the risk decreased by 83.8%. This result further confirmed the interdependent relationship between the musculoskeletal system and provided a theoretical basis for predicting the risk of sarcopenia by assessing BMD (22).

ASM and ASMI, as important indicators for measuring muscle mass, significantly reduced the risk of sarcopenia when elevated (23, 24). Maintaining muscle mass is the core of sarcopenia prevention. The higher the ASM and ASMI, the more sufficient the muscle reserve, and the lower the possibility of sarcopenia. For patients with T2DM, especially middle-aged and elderly men, while controlling blood glucose, attention should be paid to maintaining and increasing muscle mass, which can be achieved through resistance training, reasonable nutrition, etc. (25). The revised model demonstrates that sarcopenia risk can be predicted using routinely available clinical parameters without incorporating diagnostic muscle mass criteria, strength and performance. This reflects a more clinically realistic scenario where sarcopenia must be identified before DXA assessment.

In this study, a nomogram model for predicting sarcopenia was constructed based on independent influencing factors. By assigning scores to each factor, the probability of a patient’s illness can be calculated, providing a visual tool for clinical decision-making. The C-indexes of the model in the training set and the validation set were 0.773 and 0.750, respectively. The calibration curve showed a good fit between the predicted values and the true values, and the Hosmer–Lemeshow test were significant (all *p* > 0.05). The AUC of the ROC curves were 0.773 and 0.750 respectively, while these metrics alone are insufficient to confirm its clinical utility. The present study represents an essential first step in model development, but prospective impact studies are now needed to evaluate whether application of this model in clinical practice actually improves patient outcomes (e.g., through earlier sarcopenia detection or more targeted interventions). Future work should: (1) implement the model in electronic health records for real-time risk stratification, (2) assess its impact on clinical decision-making through randomized trials, and (3) evaluate long-term outcomes in patients managed using the model versus standard care. Until such studies are conducted, clinicians should interpret model predictions with appropriate caution. The sensitivity and specificity were 0.714 and 0.887 in the training set, and 0.688 and 0.796 in the validation set, indicating that the model had good discrimination, calibration, and prediction accuracy, which could balance the detection ability and specificity. The DCA demonstrated clinical utility across a wide threshold probability range (0.06–0.92), reflecting varying clinical tolerance for false-positive and false-negative predictions. The lower bound (0.06) represents scenarios where early intervention is prioritized, while the upper bound (0.92) aligns with conservative settings where overtreatment must be minimized. This range was selected to encompass diverse clinical decision-making preferences, as sarcopenia screening strategies may differ based on patient profiles and resource availability. This means that in most clinical scenarios, using this model for sarcopenia risk assessment can bring actual clinical benefits to patients and help guide clinicians to develop personalized prevention and intervention strategies. For example, for high-risk patients, measures such as intensive blood glucose management and resistance training can be taken in time to reduce the risk of sarcopenia; for low-risk patients, the monitoring frequency can be appropriately reduced to avoid waste of medical resources (26, 27).

This study has several advantages. DXA, the gold standard for evaluating muscle mass and BMD, was used to accurately measure the whole-body and local muscle and bone components, ensuring the accuracy and reliability of the data and providing high-quality data support for studying the relationship between sarcopenia and BMD. The study focused on middle-aged and elderly men with type 2 diabetes, conducting research on a specific gender and disease group, filling the gap in gender-specific research in this field. The results were more targeted and had clinical guiding significance. Not only was the relationship between sarcopenia and BMD analyzed, but also a prediction model containing multiple clinical indicators was constructed, providing a practical tool for clinical practice.

The study also had certain limitations (28, 29). First, the sample was from a single center, so its representativeness was limited. The differences in the clinical characteristics of patients from hospitals in different regions and with different medical levels may affect the external applicability of the model. Second, external validation was not carried out in this study, mainly due to time and resource limitations. Building a perfect prediction model usually requires external validation in different populations and centers to ensure its general applicability. However, due to the tight time requirements of this study and the need for more resources and cooperation to obtain external data, external validation could not be carried out. In future research, multi-center cooperation should be actively sought to further externally validate and optimize the model. Finally, the study only evaluated the indicators at a specific time point, lacking long-term follow-up data, and was unable to explore the dynamic relationship between sarcopenia and changes in BMD.

In conclusion, through DXA technology, this study found that there was a close relationship between sarcopenia and BMD in middle-aged and elderly men with type 2 diabetes. Age, HbA1c, and HOMA-IR were identified as independent risk factors, while the *T*-scores of lumbar vertebrae L1–L4 and the femoral neck were independent protective factors. The constructed nomogram model had good prediction efficacy. In the future, the sample size should be expanded, multi-center external validation should be carried out, more influencing factors should be included, and long-term follow-up should be conducted.

## Conclusion

The prediction model constructed based on DXA can effectively predict the risk of sarcopenia in middle-aged and elderly male patients with T2DM, providing a basis for clinical early screening and intervention.

## Data Availability

The original contributions presented in the study are included in the article/supplementary material, further inquiries can be directed to the corresponding author.
